# Supplementary material for: A genetic investigation in five Chinese families with keratoconus
Source: PeerJ. 2024 Sep 2;12:e18037. doi: 10.7717/peerj.18037 (PMC11376248; doi:10.7717/peerj.18037)
Supplement: Supplemental Information 1 [file peerj-12-18037-s001.docx]

**Data availability**

The datasets generated and analyzed during the current study are available in the NCBI ClinVar repository https://www.ncbi.nlm.nih.gov/clinvar/, ClinVar accession number: SCV003842308, SCV003842309, SCV003842310, SCV003842311 and SCV003842314.
