# Supplementary material for: A genetic investigation in five Chinese families with keratoconus
Source: PeerJ. 2024 Sep 2;12:e18037. doi: 10.7717/peerj.18037 (PMC11376248; doi:10.7717/peerj.18037)
Supplement: Supplemental Information 3 [file peerj-12-18037-s003.docx]

**Supplementary Table 1**. Primer sequences

| Gene | Forward | Reverse |
| --- | --- | --- |
| DOP1B | 5' ATGCGTGAGGTTCAAGGC 3' | 5' ATGGCTGTTACGAGGGTT 3' |
| HOMER3 | 5' CGAGGTTAGCAGCGAGAAG 3' | 5' CAGGGAGGTGTCCGATTGT 3' |
| IGF1R | 5' TACTCCTTCTACGTCCTCG 3' | 5' TCTGCCCACTACTGATTG 3' |
| NBEAL2 | 5' GGACAGTCCTAAGCCAACC 3' | 5' CCTACACCAATGACCACCA 3' |
| EML6 | 5' TTGTGGGCAGACTCAGGG 3' | 5' AAGGGTTCAAACATGCAAAGA 3' |
