# Supplementary figures and images for: A genetic investigation in five Chinese families with keratoconus
Source: PeerJ. 2024 Sep 2;12:e18037. doi: 10.7717/peerj.18037 (PMC11376248; doi:10.7717/peerj.18037)

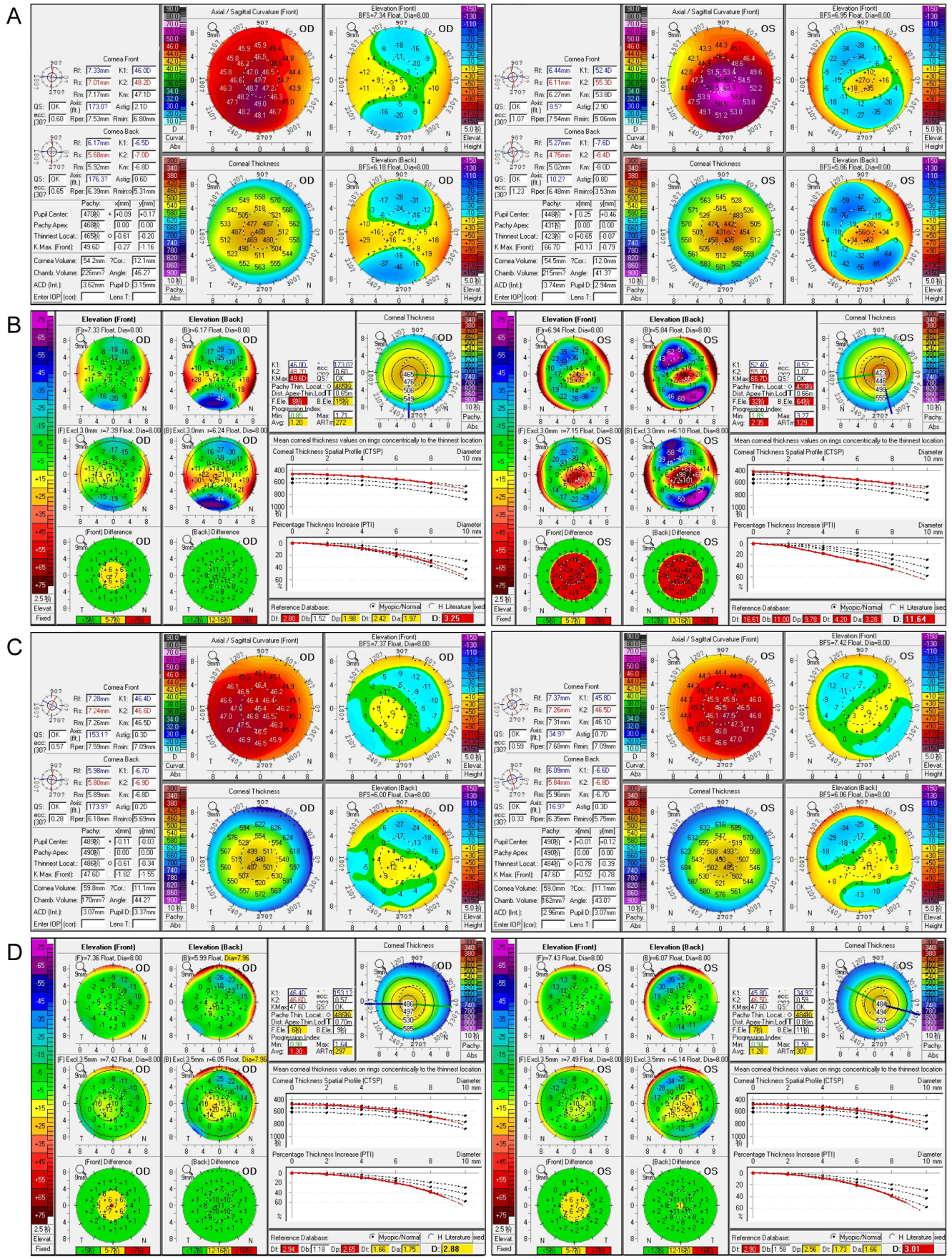

Supplement: Supplemental Information 4 — 1A: The proband, apparent inferior steepening and irregular astigmatism were shown, especially in the left eye (OS). Maximum anterior surface curvature (MASC) was 49.6D in the right eye and 66.7D in the left eye. The means of posterior elevation of the cornea (PCE) were 16 µm (right eye) and 62 µm (left eye). The central corneal thicknesses (CCT) were 465 and 423 µm in the right and left eye, respectively. 1B: Belin analysis of II.1 showed that the corneal thickness progression deviation (Dp) values in both eyes were in a suspicious value state (1.98 in the right and 9.78 in the left eye); Pentacam tomographic composite index (BAD-D) demonstrated a marked pathological change in both eyes (3.25 in the right eye and 11.64 in the left eye). 1C: subject I.1, MASC was 44.0D in the right eye and 44.8D in the left eye, PCE values at the thinnest point of the cornea were greater than 13 μm in both eyes (18 µm in the right eye and 14 µm in the left eye), CCT were 578 and 577 µm in the right and left eyes, respectively. 1D: Belin analysis of I.1 showed that the Dp values in both eyes were in a suspicious value state (2.65 in the right and 2.56 in the left eye), BAD-D demonstrated a marked pathological change in both eyes (2.88 in the right eye and 3.01 in the left eye). [file peerj-12-18037-s004.pdf]

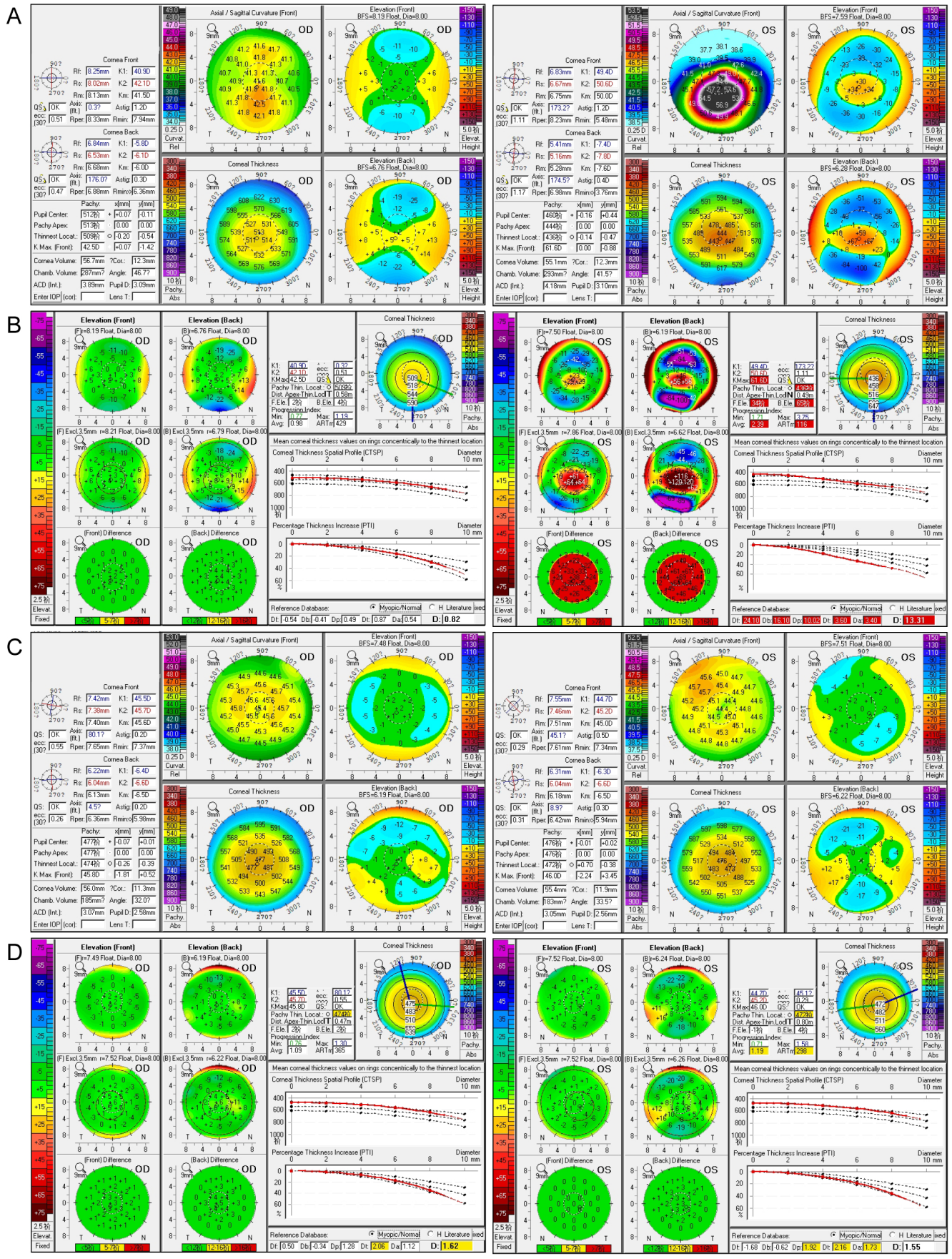

Supplement: Supplemental Information 5 — 2A: the proband (II.1), maximum anterior surface curvature was 42.5D in the right eye and 61.6D in the left eye, the means of PCE and CCT were 61 and 481 µm in the right eye and 67 and 436 µm in the left eye, respectively. 2B: Belin analysis of II.1, the corneal thickness progression deviation (Dp) value in the left eye was 10.02, Pentacam tomographic composite index (BAD-D) was 13.31. 2C: The mother of the proband suffered from central cornea thinning, CCTs were 474 µm in the right eye and 472 µm in the left eye. 2D: Belin analysis of the mother showed that the Dp value in the left eye was in a suspicious value state (1.28 in the right eye and 1.92 in the left eye). BAD-D demonstrated a pathological change in the right eye (1.62 in the right eye and 1.55 in the left eye). [file peerj-12-18037-s005.pdf]

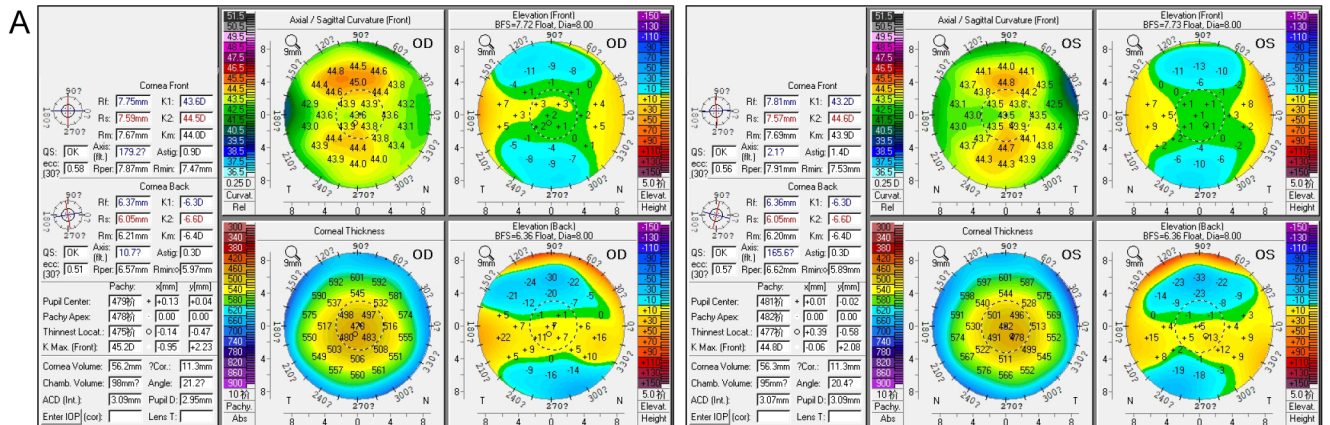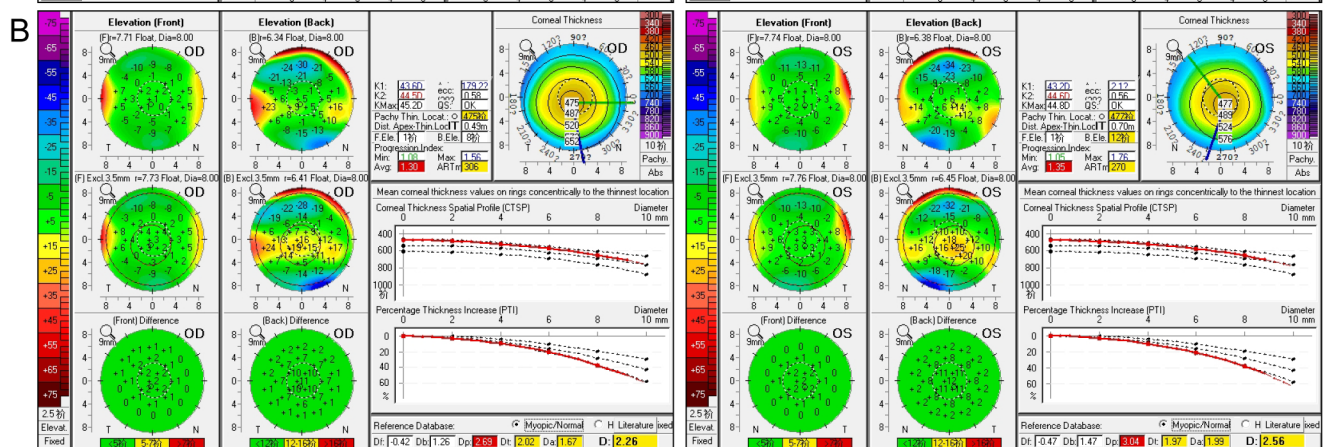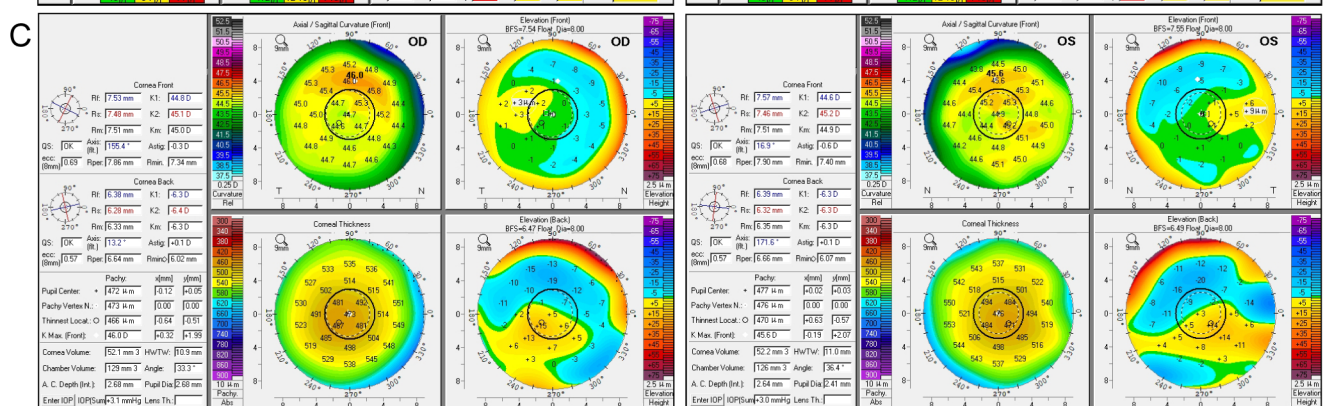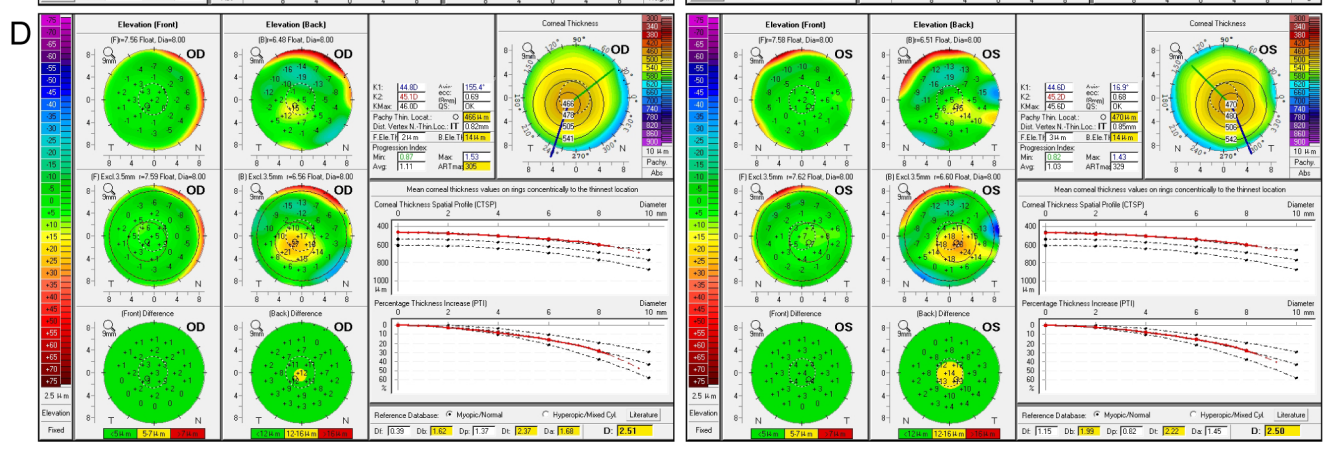

Supplement: Supplemental Information 7 — 4A: the proband (II.1), maximum anterior surface curvature (MASC) was 45.2D in the right eye and 44.8D in the left eye, the means of PCE and CCT were 11 and 475 µm in the right eye and 13 and 477 µm in the left eye, respectively. 4B: Belin analysis of proband showed that the corneal thickness progression deviation (Dp) values in both eyes were in a suspicious value state (2.69 in the right and 3.04 in the left eye), Pentacam tomographic composite index (BAD-D) demonstrated a pathological change in both eyes (2.26 in the right eye and 2.56 in the left eye). 4C: I.2, mother of the proband, showed central cornea thinning, CCTs were 466 µm in the right eye and 470 µm in the left eye, PCE values at the thinnest point of the cornea were greater than 13 μm in both eyes (15 µm in the right eye and 14 µm in the left eye). 4D: Belin analysis of I.2, BAD-D demonstrated a pathological change in both eyes (2.51 in the right eye and 2.50 in the left eye). [file peerj-12-18037-s007.pdf]

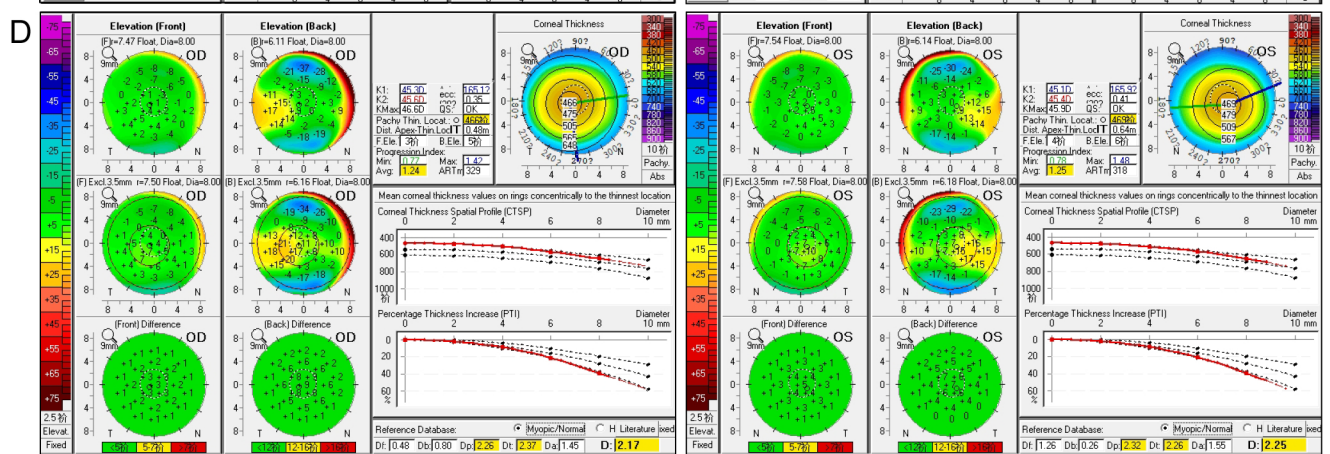

Supplement: Supplemental Information 8 — 5A: Proband (II.1). MASCs were 44.2 D in the right eye and 63.3 D in the left eye. The means of PCE and CCT were 13 and 500 µm in the right eye and 73 and 460 µm in the left eye, respectively. 5B: Belin analysis showed that Dp values in both eyes were in a suspicious state (3.02 in the right and 9.70 in the left eye). BAD-D demonstrated a pathological change in both eyes (2.90 in the right eye and 9.88 in the left eye). 5C: subject II1, maximum anterior surface curvature was 46.6 D in the right eye and 45.9 D in the left eye. The posterior surface elevation values at the thinnest point of the cornea were in a normal range in both eyes (5 µm in the right eye and 6 µm in the left eye). She suffered central cornea thinning. CCTs were 466µm in right eye and 469 µm in left eye, 5D: Belin analysis of subject II1 showed that the corneal thickness progression deviation (Dp) values in both eyes were in a suspicious value state (2.26 in the right and 2.32 in the left eye), Pentacam tomographic composite index (BAD-D) demonstrated a pathological change in right eye (2.17 in the right eye and 2.25 in the left eye). [file peerj-12-18037-s008.pdf]
