# Supplementary material for: A genetic investigation in five Chinese families with keratoconus
Source: PeerJ. 2024 Sep 2;12:e18037. doi: 10.7717/peerj.18037 (PMC11376248; doi:10.7717/peerj.18037)
Supplement: Supplemental Information 9 — (a) Regional plots for variants in IGF1R. Twenty variants reached the corrected P at 0.05. LD was calculated in these variants. (b) Regional plots for variants in EML6. Fifteen sites reached the corrected P at 0.05. LD was calculated in these variants. (c) Regional plots for variants in DOP1B. Five sites reached the corrected P at 0.05. LD was calculated in these variants. None variations in HOMER3 (d) and NBESL2 (e) reached the significance threshold, i.e., corrected P at 0.05. [file peerj-12-18037-s009.pdf]

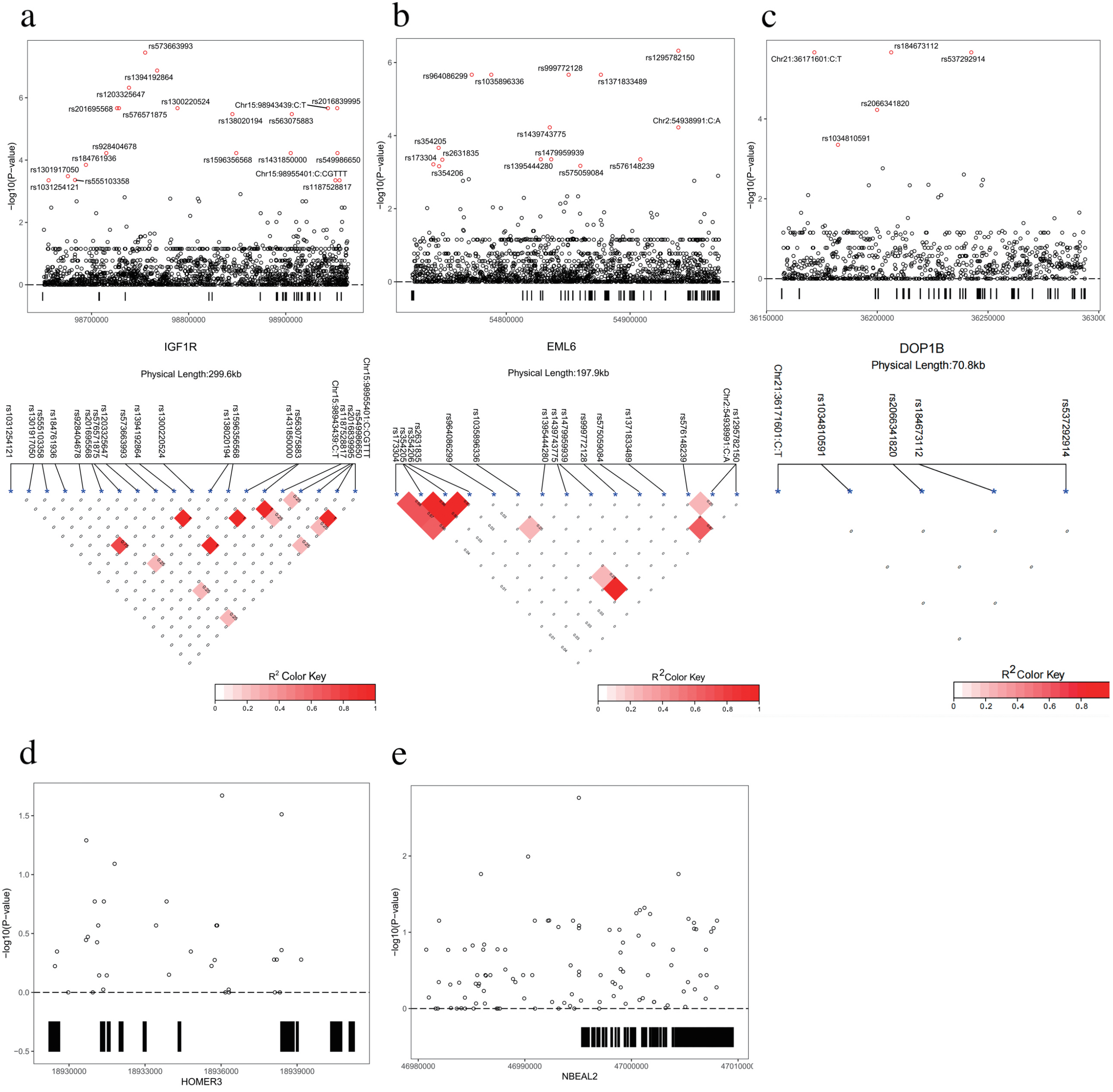
a
b
c
d
e
−log10(P−value)
rs573663993
rs1394192864
rs1203325647
rs201695568
rs576571875
rs1300220524
Chr15:98943439:C:T
rs2016839995
rs138020194
rs563075883
rs928404678
rs184761936
rs1301917050
rs555103358
rs1031254121
rs1596356568
rs1431850000
rs549986650
Chr15:98955401:C:CGTTT
rs1187528817
98700000
98800000
98900000
IGF1R
Physical Length:299.6kb
rs1295782150
rs999772128
rs964086299
rs1035896336
rs1371833489
Chr2:54938991:C:A
rs1439743775
rs354205
rs2631835
rs173304
rs354206
rs1479959939
rs1395444280
rs575059084
rs576148239
54800000
54900000
EML6
Physical Length:197.9kb
rs184673112
Chr21:36171601:C:T
rs537292914
rs2066341820
rs1034810591
36150000
36200000
36250000
DOP1B
Physical Length:70.8kb
R2 Color Key
18930000
18933000
18936000
18939000
HOMER3
46980000
46990000
47000000
NBEAL2
