# Supplementary material for: A genetic investigation in five Chinese families with keratoconus
Source: PeerJ. 2024 Sep 2;12:e18037. doi: 10.7717/peerj.18037 (PMC11376248; doi:10.7717/peerj.18037)
Supplement: Supplemental Information 6 — 3A: The proband (II.1), maximum anterior surface curvature (MASC) was 47.0D in the right eye and 55.1D in the left eye. The PCEs were 8 µm (right eye) and 31 µm (left eye). The CCTs were 525 and 490 µm in the right and left eye, respectively. 3B: Belin analysis of II.1 showed that the corneal thickness progression deviation (Dp) values in both eyes were in a suspicious value state (2.36 in the right and 6.66 in the left eye), Pentacam tomographic composite index (BAD-D) demonstrated a marked pathological change in left eye (1.59 in the right eye and 6.61 in the left eye). 3C: subject I.2, father of the proband, MASC was 45.6D in the right eye and 45.5 D in the left eye, CCTs were 484 µm in right eye and 475 µm in left eye. 3D: Belin analysis of I.2, showed that the Dp values in both eyes were in a suspicious value state (2.28 in the right and 1.99 in the left eye), BAD-D demonstrated a pathological change in the right eye (2.09 in the right eye and 1.59 in the left eye). [file peerj-12-18037-s006.pdf]

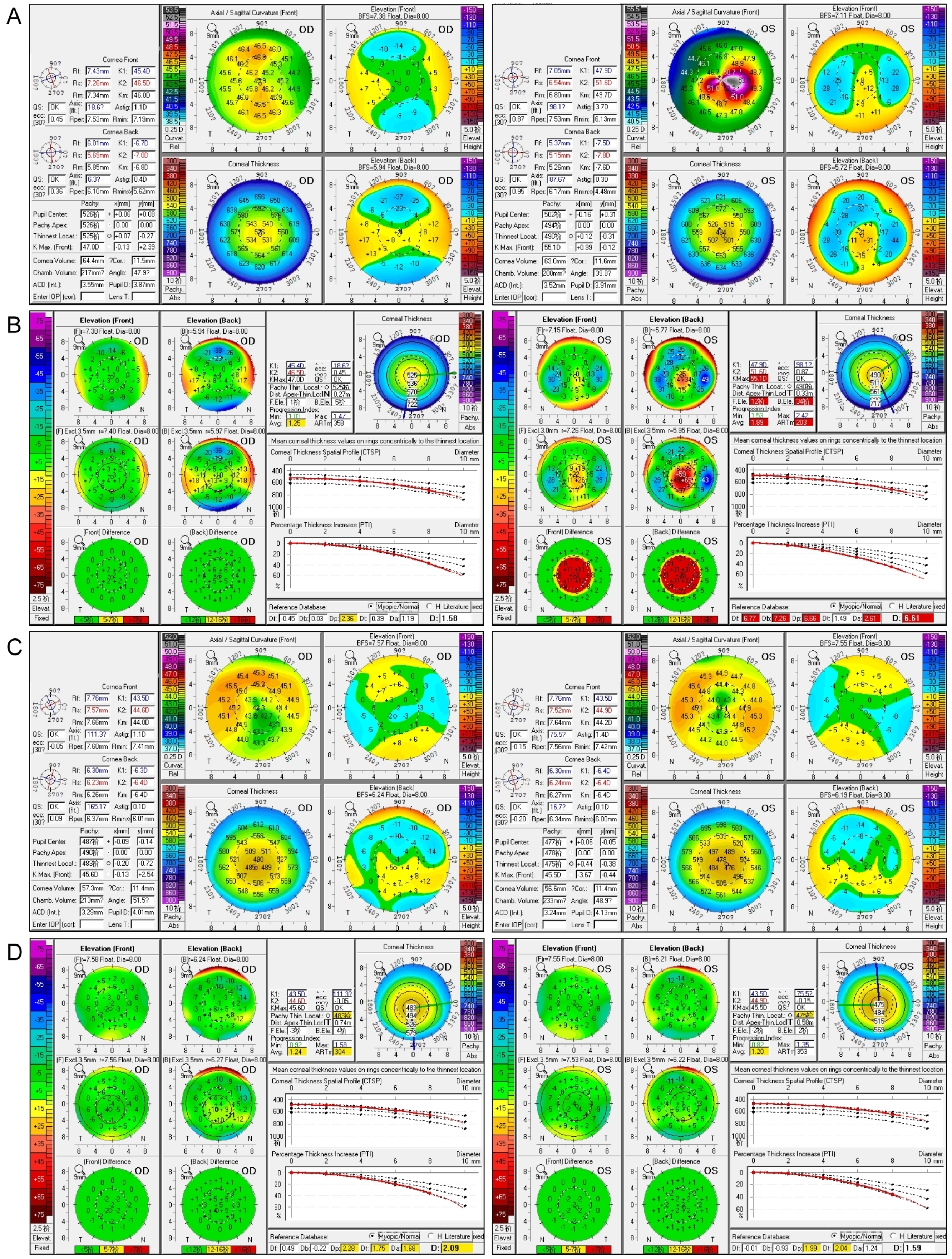
A
Axial / Sagittal Curvature (Front)
Elevation (Front)
Corneal Thickness
Elevation (Back)
Cornea Front
Cornea Back
OD
OS
B
Elevation (Front)
Elevation (Back)
Corneal Thickness
(Front) Difference
(Back) Difference
Mean corneal thickness values on rings concentrically to the thinnest location
Corneal Thickness Spatial Profile (CTSP)
Percentage Thickness Increase (PTI)
Reference Database:
Myopic/Normal
D: 1.58
D: 6.61
C
Axial / Sagittal Curvature (Front)
Elevation (Front)
Corneal Thickness
Elevation (Back)
D
Elevation (Front)
Elevation (Back)
Corneal Thickness
(Front) Difference
(Back) Difference
Corneal Thickness Spatial Profile (CTSP)
Percentage Thickness Increase (PTI)
D: 2.09
D: 1.59
